# Perceptions of novel warnings compared with current warning on tobacco packs, and warnings on cigarette sticks: A cross-sectional survey of college students in Southern India

**DOI:** 10.18332/tid/160082

**Published:** 2023-04-28

**Authors:** Somya Mullapudi, Muralidhar M. Kulkarni, Veena G. Kamath, John Britton, Crawford Moodie, Asha Kamath

**Affiliations:** 1 Department of Community Medicine, Kasturba Medical College, Manipal Academy of Higher Education, Manipal, India; 2 UK Centre for Tobacco & Alcohol Studies, Faculty of Medicine & Health Sciences, University of Nottingham, Nottingham, England; 3 Institute for Social Marketing and Health, University of Stirling, Stirling, United Kingdom; 4 Department of Data Science, Prasanna School of Public Health, Manipal Academy of Higher Education, Manipal, India

**Keywords:** survey, packaging, cigarettes, warnings

## Abstract

**INTRODUCTION:**

In India there is insufficient knowledge of the risks associated with tobacco use. Increasing awareness of these risks is critical, with pictorial warnings on tobacco packs a cost-effective way to communicate this information. We explored perceptions of the current warning, ‘Tobacco causes cancer’, displayed on packs in India and four novel warnings about other potential impacts of tobacco use including social, financial, and environmental, but also complications with diabetes. As loose cigarette sales are common in India, we also explored perceptions of warnings on cigarette sticks.

**METHODS:**

A cross-sectional survey of college students aged ≥18 years in Karnataka, India, was conducted between January 2019 and February 2020. Participants were asked about salience, believability, and cognitive processing of warnings currently on packs. They were then shown an image of one current and four novel warnings and asked about their perceived effectiveness in preventing uptake and reducing and stopping tobacco use. They were then asked about warnings on cigarette sticks.

**RESULTS:**

Most participants (70.2%) recalled warnings on packs and considered them believable (55.7%), but only 12.0% read and 12.4% thought about them often. Warnings about the health impacts of tobacco use were viewed as most effective in preventing uptake, and reducing and stopping tobacco use. Nevertheless, at least a third of participants rated warnings pertaining to financial, social, and environmental impacts effective in preventing uptake, and reducing and stopping tobacco use. Approximately one-fifth (22.0%) thought that warnings on cigarette sticks would deter initiation.

**CONCLUSIONS:**

Our results suggest that health warnings are perceived as most effective in discouraging tobacco use among college students in Karnataka. While viewed as less effective than health warnings, novel non-health related messages were viewed as effective in preventing uptake, and reducing and stopping tobacco use by at least one in three participants. Warnings on cigarette sticks may help complement warnings on cigarette packs.

## INTRODUCTION

Morbidity and mortality due to tobacco use are highest in low- and middle-income countries (LMICs), home to 80% of global smokers^1^ and most smokeless tobacco users^2^. With more than 1.35 billion people and an increasingly affluent middle class, India presents a major market opportunity for tobacco companies^3^. While the prevalence of tobacco use in India has fallen, from 34.6% in 2008 to 28.6% in 2016–2017^4-5^, the absolute number of tobacco users remains high^4^. The mean age of initiation of tobacco use (both for cigarettes and smokeless tobacco) is 18.9 years, highlighting the need to target this age group^5-6^.

Knowledge of the risks of tobacco use is lower in LMICs than elsewhere^7^. Communication of these risks is fundamental to reducing tobacco use, with pictorial warnings on tobacco packs viewed as the most cost-effective means of educating consumers on these risks^1,8^. The guidelines for Article 11 of the Framework Convention on Tobacco Control encourage Parties to use a range of warnings as different messages resonate with different audiences^9^. Besides health, warnings on the social, environmental and economic impacts of tobacco use are recommended^9^. However, in India, the current two pictorial warnings on packs depict end-stage issues like death and cancer, with a paucity of research on alternative health-related and non-health-related messages^10-11^.

The widespread practice of selling loose cigarettes in India^12^ insulates many consumers from exposure to on-pack warnings when they purchase or use cigarettes. The absence of warnings on loose cigarettes may lead consumers to underestimate the harm. Indeed, some states, such as Karnataka (where this study was conducted), have banned single sales, as cigarette sticks do not display a warning^13^; however, the enforcement of the law is weak. A growing number of studies have explored how the cigarette stick could be used to communicate the risks of smoking, for instance, by displaying a warning on the cigarette paper. Most have explored responses to the warning ‘Smoking kills’, finding that it has reduced appeal, increased perceptions of harm, and is considered off-putting^14-23^. However, all research has been conducted in high-income countries, with no studies in countries where the sale of single cigarettes is common.

To address these gaps, we explored the perceived effectiveness of one health warning currently displayed on packs in India and four novel warnings, as well as warnings on individual cigarettes, among college students in India.

## METHODS

### Design and sample

A cross-sectional survey was conducted, between January 2019 and February 2020, with college students aged ≥18 years from the Udupi district of Karnataka in Southern India. A list of all undergraduate colleges in the study area was obtained by searching university websites served as a sampling frame. The colleges were then stratified into ‘Technical’, ‘Health Science’ and ‘Other’ streams. The ‘Other’ category included studies such as Bachelor of Commerce, Bachelor of Arts, Bachelor of Social Sciences, and Bachelor of Law. Of the 62 colleges in Udupi district, 30 were randomly selected, with 29 agreeing to participate. Considering the proportion of individuals who interpreted the health warnings correctly to be 25.5% with a relative precision of 12.5%, cluster effect of two, the sample size was calculated to be 1495. Based on an estimated non-response rate of up to 20%, a total of 1794 students were required. The total number of students approached (i.e. who were given an information sheet on the first visit to each college) was 1894. Of these 1894 students, 1788 students were present on the day the questionnaires were distributed, with two declining to participate and 30 returning incomplete questionnaires, leaving 1756 completed surveys (92.7% response rate).

Principals of the selected colleges were contacted by phone to seek approval to conduct the study and obtain information about the number of potentially eligible students. All colleges were visited twice, first to distribute the information sheet and second, within 2–3 days of the first visit, to administer (printed) questionnaires; these were administered to students based on their language preference (either English or Kannada, the local language). Students present during the day of the survey were included if they provided informed consent. They were given 30–45 minutes to complete the pre-tested questionnaire. After the completed questionnaires were collected, a pamphlet with information on the harmful effects of tobacco use was distributed to each student. Prior to the survey, a pilot was conducted with 100 students to pretest the data collection methodology and refine the questionnaire.

**Figure 1:**
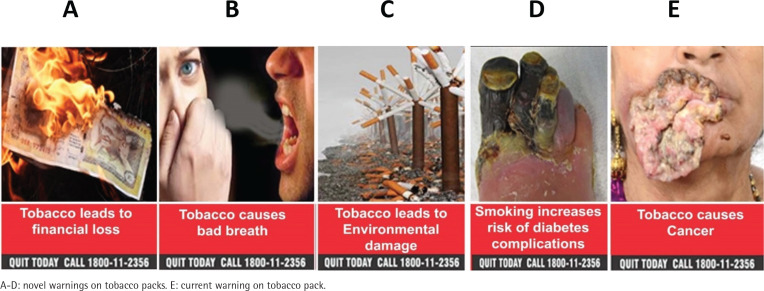
Novel and current tobacco pack warnings

**Figure 2:**
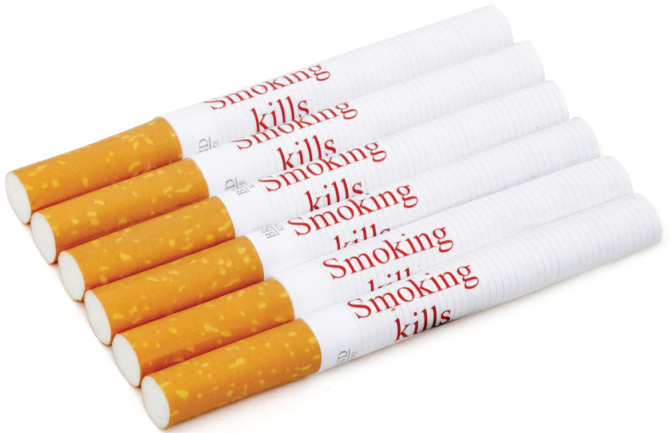
Warnings on individual cigarettes

The survey captured sociodemographic information, knowledge about the harms of tobacco use, ever and current tobacco use, and perceptions of existing and novel warnings on packs, and warnings on individual cigarettes. For the pack warnings, we created four novel warnings for India, drawn from the WHO Database^24-27^, with impact: 1) financial loss, 2) bad breath, 3) environmental harm, and 4) diabetes (Figure 1). These were tested against one of the current warnings, ‘Tobacco causes cancer’. The warning on the paper of cigarette sticks was ‘Smoking kills’ (Figure 2), consistent with past research^14-23,28^.

### Measures

*Sociodemographics*

We collected data on age, gender, subject studied, religion and socioeconomic status, using the modified Kuppuswamy socioeconomic scale^29^.

*Knowledge about the harms of tobacco use*

This was assessed by asking if tobacco use is harmful to health, harmful to be near a person who is smoking tobacco, and whether quitting has a positive impact on health, with response options for each ‘yes’, ‘no’, and ‘not sure’. They were also given a list of eight health issues (cancer, lung diseases, heart disease, paralysis, diabetes, blindness, hearing loss, and fertility problems) and asked to indicate those that they believed were associated with tobacco use. A knowledge of harms score was calculated by assigning one point for each correct response (‘yes’ to the first three questions, and for every health issue indicated). The cumulative score was then divided into high (range: 8–11), medium (range: 6–7), and low (range: 0–5).

*Tobacco use*

Ever use was defined as any lifetime tobacco use, current use as use of tobacco within the last 30 days, and never use as no lifetime tobacco use^30^.

*Unaided recall of warnings*

Participants were asked: ‘On thinking about the health warnings you have seen on tobacco products, what message or picture do you remember most?’. Responses were recorded as correct if they matched the warning themes.

*Warning salience and depth of processing*

Participants were asked: ‘In the last 30 days how often, if at all, have you read or looked closely at the warnings on packs’, and ‘In the last 30 days how often, if at all, did you think about what the warnings on packs are telling you?’. Response options for both were categorized as ‘often’ and ‘very often’ versus ‘never’, ‘rarely’, ‘sometimes’ and ‘don't know’.

*Believability*

Participants were asked: ‘How believable do you think warnings are?’, Response options were: ‘not at all believable’, ‘a little believable’, ‘somewhat believable’, ‘very believable’, ‘extremely believable’ and ‘don’t know’. Responses ‘very believable’ and ‘extremely believable’ were recoded as ‘believable’, and the remaining options as ‘not believable’.

*Perceptions of the effectiveness of pack warnings*

Participants were shown an image of one of the current warnings on packs and four novel warnings (Figure 1) and asked about the effectiveness of each in reducing uptake, reducing tobacco use, and stopping tobacco use. Each item was measured on the categorial scale: 1–3 = ‘ineffective’; 4 = ‘neither effective nor ineffective’; and 5–7 = ‘effective’.

*Perceptions of warnings on cigarette sticks*

Participants were shown an image of warnings on individual cigarettes (Figure 2) and asked whether a warning on each cigarette stick would put people off starting to smoke, make people want to give up smoking, and support for warnings on sticks. All three questions were rated on a 5-point scale. The item on giving up was reverse coded at the analysis stage so that a high score reflected a negative reaction. The items on putting people off starting to smoke and support for warnings on sticks were then dichotomized into those who had a positive reaction (scores 1 and 2) versus those who had neutral or negative reactions (scores 3 to 5). Participants were also asked the extent to which a warning on each cigarette would make them think about the health risks of smoking. Response options, ‘not at all’, ‘a little’, ‘somewhat’ were compared with ‘a lot’, with ‘don't know’ and ‘no response’ considered missing values.

To assess perceived willingness to try, participants were shown an image of a cigarette with and without a warning and asked the likelihood of trying each if offered to them by a friend, with responses from 1 = ‘not at all likely’ to 7 = ‘very likely’. This was dichotomized into those who indicated that they were more unlikely to try a cigarette (scores: 1 to 4) versus those who were more likely to try a cigarette (scores: 5 to 7).

### Statistical analysis

Data were entered in Microsoft excel 2007 and exported to and analyzed using SPSS version15. Frequencies and percentages were calculated for the selected variables. Factors influencing perceptions of warnings on tobacco packs were analyzed among tobacco ever users compared to never users, using logistic regression. Factors influencing the association of perceptions of warnings on cigarette sticks and smoking status were analyzed using logistic regression.

## RESULTS

### Sample characteristics

Of the 1756 participants, approximately a third were from each college stream (34.5% health science, 32.7% technical, and 32.8% other). Mean age was 19.3 years (SD=1.1), with most participants female (60.4%). One-sixth (17.5%) had ever used tobacco, with 6.4% current smokers. Most current smokers (74.5%) normally purchased loose cigarettes or bidis.

### Salience, believability and cognitive processing of warnings on tobacco packs

In the past 30 days, most participants (80.0%) reported ‘never, rarely or sometimes’ noticing warnings on packs, with 76.3% ‘never, rarely or sometimes’ thinking about them. Approximately three-fifths (55.7%) of the participants considered warnings ‘very’ or ‘extremely’ believable (Table 1). Univariate analysis found that tobacco ever users had higher awareness, salience, and depth of processing of warnings on tobacco packs compared to never users, while they were less likely to believe the warnings. Multivariate analysis found that tobacco ever users were less likely to believe the warnings on tobacco packs after adjusting for significant sociodemographic variables (AOR=1.6; 95% CI: 1.2–2.1) (Table 2).

**Table 1:** Salience, believability and cognitive processing of the current health warnings on tobacco packs, among undergraduate college students in Udupi district (N=1756)

*Characteristics*	*n (%)*
**Salience** (reading/looking closely at warnings)	
Often/very often	211 (12.0)
Never/rarely/sometimes/don’t know	1405 (80.0)
No response	140 (8.0)
**Believability** (believability of warnings)	
Very/extremely	978 (55.7)
Not at all/a little/somewhat/don’t know	626 (35.6)
No response	152 (8.7)
**Cognitive processing** (thinking about warnings)	
Often/very often	218 (12.4)
Never/rarely/sometimes/don’t know	1339 (76.3)
No response	199 (11.3)

**Table 2:** Association of knowledge about tobacco use, awareness, recall, salience, depth of processing, and believability of the health warnings with ever tobacco use among undergraduate college students in Udupi district (N=302)

*Variable*	*Total (N=1725)*	*Ever tobacco user (N=302) n (%)*	*OR (95% CI)*	*p*	*AOR (95 % CI )*	p*
**Knowledge of harms of tobacco use**				0.547		
High (Ref.)	414	82 (19.8)	1			
Medium	875	151 (17.3)	0.9 (0.6–1.2)			
Low	375	62 (16.5)	0.9 (0.6–1.2)			
No response	61					
**Awareness of HW**				<0.001		0.103
Yes (Ref.)	1501	282 (18.8)	1		1	
No	201	19 (9.5)	0.4 (0.3–0.7)		0.6 (0.4–1.1)	
No response	23					
**Unaided recall of HW**				0.117		
Yes (Ref.)	1214	225 (18.5)	1			
No	511	77 (15.1)	0.8 (0.6–1.1)			
**Salience of HW**				<0.001		0.052
Yes (Ref.)	209	55 (26.3)	1		1	
No	1382	233 (16.9)	0.5 (0.4–0.8)		0.7 (0.5–1.0)	
No response	134					
**Depth of processing of HW**				0.004		0.072
Yes (Ref.)	216	57 (26.4)	1		1	
No	1318	230 (17.5)	0.6 (0.4–0.9)		0.7 (0.5–1.0)	
No response	191					
**Believability of HW**				<0.001		0.003
Yes (Ref.)	964	147 (15.2)	1		1	
No	617	142 (23.0)	1.6 (1.3–2.1)		1.6 (1.2–2.1)	
No response	144					

HW: health warning. AOR: adjusted odds ratio: after adjusting for gender, religion, and socio-economic quintiles.

* p<0.05.

### Perceptions of current and novel warnings

Warnings on packs were recalled, unaided, by 1232 (70.2%) participants. The current warning on packs was rated the most effective in preventing uptake (85.5%), reducing tobacco use (75.1%) and stopping tobacco use (73.5%), followed by the warning about diabetes complications. The warnings pertaining to financial loss, social impact and environmental impact were considered less effective, but nevertheless, between 43% and 45% considered them effective in preventing uptake, and between 32% and 35% considered them effective in reducing and stopping tobacco use (Table 3).

**Table 3:** Perceptions of the effectiveness of novel warnings* and current warning on preventing, reducing and stopping tobacco use among undergraduate college students in Udupi district (N=1756)

*Perceived effectiveness*	*Effective*	*Neither effective nor ineffective*	*Ineffective*
	*n (%)*	*n (%)*	*n (%)*
**Preventing uptake**			
Tobacco leads to financial loss	795 (45.3)	178 (10.1)	708 (40.3)
Tobacco causes bad breath	769 (43.8)	177 (10.1)	717 (40.8)
Tobacco leads to environmental damage	774 (44.1)	184 (10.5)	684 (39.0)
Smoking increases risk of diabetes complications	1256 (71.5)	172 (9.8)	231 (13.2)
Tobacco causes cancer	1501 (85.5)	53 (3.0)	115 (6.5)
**Reducing tobacco use**			
Tobacco leads to financial loss	626 (35.6)	180 (10.3)	855 (48.7)
Tobacco causes bad breath	581 (33.1)	225 (12.8)	824 (46.9)
Tobacco leads to environmental damage	587 (33.4)	215 (12.2)	824 (46.9)
Smoking increases risk of diabetes complications	1095 (62.4)	209 (11.9)	326 (18.6)
Tobacco causes cancer	1319 (75.1)	127 (7.2)	207 (11.8)
**Stopping tobacco use**			
Tobacco leads to financial loss	629 (35.8)	172 (9.8)	851 (48.5)
Tobacco causes bad breath	565 (32.2)	224 (12.8)	842 (47.9)
Tobacco leads to environmental damage	596 (33.9)	220 (12.5)	810 (46.1)
Smoking increases risk of diabetes complications	1100 (62.6)	180 (10.3)	345 (19.6)
Tobacco causes cancer	1291 (73.5)	113 (6.4)	251 (14.3)

* The first four warnings listed are the novel tobacco pack warning messages, the fifth related to cancer is the current warning message.

### Perceptions of warnings on individual cigarettes

Approximately one-fifth of participants indicated that a warning on cigarette sticks would put people off starting to smoke (22.0%), with no significant difference by smoking status. Almost one-third of participants felt that the cigarette stick warnings would make people want to quit smoking (32.1%), with no significant difference in smoking status. One-third reported that it would make them think a lot about the risks of smoking (33.8%), with never smokers more likely than ever smokers to think about the health risks of smoking (AOR=1.6; 95% CI: 1.2– 2.2, p=0.005). More than one-third of participants supported a warning on individual cigarettes (35.5%), with no significant difference by smoking status (Table 4 and Supplementary file Table 1).

**Table 4:** Perceptions of warnings on cigarette sticks, among undergraduate college students in Udupi district (N=1756)

*Perceptions*	*Total*	
	*n*	*%*
(1) Would put people off smoking*	214	12.2
(2)	172	9.8
(3)	279	15.9
(4)	196	11.2
**(5) Would not put people off smoking**	317	18.1
Don’t know/no response	578	32.8
(1) Would not make people want to give up smoking*	196	11.2
(2)	177	10.1
(3)	262	14.9
(4)	236	13.4
**(5) Would make people want to give up smoking**	328	18.7
Don’t know/no response	557	31.7
(1) All cigarettes should have a warning on them*	554	31.5
(2)	70	4.0
(3)	108	6.2
(4)	106	6.0
**(5) All cigarettes should not have a warning on them**	493	28.1
Don’t know/no response	425	24.2
**Warning makes you think about health risks**		
Not at all	73	4.2
A little	184	10.5
Somewhat	479	27.3
A lot	595	33.8
Don’t know/no response	425	24.2

* Response options are on a scale of 1 to 5, with the item on ‘wanting to quit’ reverse coded so that a high score reflects a negative reaction.

The likelihood of trying a cigarette, if offered by a friend, was 14.8% for the stick without a warning and 8.6% for the stick with a warning. Never smokers were less likely than ever smokers to indicate that they would try a regular cigarette if offered one by a friend (AOR=0.2; 95% CI: 0.2–0.3, p<0.001), and less likely to try a cigarette with a warning (AOR=0.3; 95% CI: 0.2–0.4, p<0.001) (Supplementary file Table 1).

## DISCUSSION

Among undergraduate college students in the Udupi district of India, we found that regardless of tobacco use, warnings about the health impacts of tobacco use, particularly the current warning on packs about cancer, are viewed as most effective in preventing uptake and reducing or stopping tobacco use. Novel warnings about the financial, environmental and social impacts of tobacco use were considered less effective, but nevertheless rated as effective in preventing uptake and reducing or stopping tobacco use by at least a third of participants. In what is, to our knowledge, the first study exploring perceptions of warnings on cigarettes in a market where the sale of single cigarettes is the norm, we found that one-fifth of participants thought that they would prevent uptake, with approximately one-third indicating they would make them think of risks of smoking, help smokers quit or would support them. We also found that while approximately one in seven (14.8%) participants indicated that they would be more likely than not to smoke a regular cigarette if offered by a friend, one in eleven (8.6%) indicated that they would do so if the cigarette stick displayed a warning.

Most (70%) participants in our study recalled warnings on packs, consistent with a study in Karnataka conducted shortly after the larger warnings (covering 85% of the main display areas) appeared on packs^31^, but much higher than in research prior to larger warnings being required, where awareness was less than 40%^32^. The current warning on packs concerning cancer was rated as most effective in preventing uptake, and reducing or stopping tobacco use, with the novel warning about diabetes complications also viewed as a strong deterrent. While the warnings on the financial, social and environmental impacts of tobacco use had lower ratings, but more than two-fifths of participants thought these would effectively prevent uptake. As only a very small number of warnings are used on packs in India, increasing the likelihood of desensitization, novel warnings may be appropriate within a large set of warnings. Given that there remains a lack of research on non-health warnings^11^, despite being recommended by the FCTC^9^, further work on what other messages may resonate with young people is needed.

The sale of single cigarettes offers an inexpensive route to smoking and allows smokers to consume tobacco even when financially constrained. Despite single cigarette sales being banned in Karnataka, smokers routinely purchase cigarettes or bidis, meaning that they are not necessarily exposed to pack warnings during purchase and consumption. There are penalties for selling single cigarettes in Karnataka, and a mobile app called ‘Stop Tobacco’ was launched by the State anti-tobacco cell in all districts (including Udupi) to allow consumers to report violations of this or any other infringements of the Cigarette and Other Tobacco Product Act^33^. However, these measures by themselves are insufficient to deter this practice. One-third of participants felt warnings on cigarette sticks would make them think of the risks of smoking. Although not directly comparable, qualitative research in the UK with adult smokers and marketing experts found warnings on sticks to be considered a constant reminder of the health risks^16,20^. Participants in India were less likely to think that a warning on each stick would stop people from starting, make people want to give up smoking, and support such a measure than was found in research with children and young adults in the UK^15-20,22-23,28,34^. Qualitative research in India exploring possible reasons for this discrepancy would be of value.

### Limitations

The study has some limitations. As a cross-sectional survey with college students, the results are not generalizable to the wider population of India. The novelty of the warnings on packs and sticks, and brief exposure through the questionnaire, may have influenced responses. Although there were a relatively small number of tobacco users in the sample, the pack warnings are intended to encourage users to reduce consumption and quit and discourage non-users from starting. Concerning perceptions of warnings on a cigarette stick, we did not assess differences by susceptibility among never smokers, or quit intentions or attempts among smokers. Despite these limitations, our work is innovative, given the focus on novel warnings on packs and warnings on cigarette sticks for the first time in an LMIC. Further quantitative and qualitative research on warnings on sticks in India and other LMICs, particularly with young people and children and where single cigarette sales are common, may help inform future pack or stick policy changes aimed at strengthening tobacco control efforts.

## CONCLUSIONS

Among our sample of predominantly tobacco non-using university students in the Udupi district of India, warnings about the health impacts of tobacco use were viewed as most effective in preventing uptake than reducing or stopping tobacco use. However, novel warnings about the financial, environmental and social impact of tobacco use may have a place within a larger warning set, especially for those that downplay or ignore the health risks or are more concerned about non-health impacts. The large-scale selling of loose cigarettes in India makes warnings on cigarette sticks a possible strategic tool to deter use of these products.

## Data Availability

The data supporting this research are available from the authors on reasonable request.
