# Supplementary material for: Perceptions of novel warnings compared with current warning on tobacco packs, and warnings on cigarette sticks: A cross-sectional survey of college students in Southern India
Source: Tob Induc Dis. 2023 Apr 28;21:50. doi: 10.18332/tid/160082 (PMC10141829; doi:10.18332/tid/160082)
Supplement: Supplementary file 1 [file TID-21-50-s1.pdf]

Supplementary Table 1: Logistic regression of association between views on cigarette warnings and smoking status, demographic and environmental factors

Variables	**Would put people off starting to smoke** 1=Put people off starting (1-2) (386), 0=Not put people off starting (3-5)(792).			**Would make people want to give up smoking** 1=Make people give up (4-5) (564), 0=Not make people give up (1-3)(635).			**All cigarettes should have a warning on them** 1=Should have warnings (1-2) (624), 0=Should not have warnings (3-5)(707).			**Warning makes you think about health risks** 1=A lot (595), 0=Not at all, A little, Somewhat (736).			**Likelihood of trying if a friend offered cigarette without warning** 1=More likely (5-7) (260), 0=More unlikely (1-4) (1314).			**Likelihood of trying if a friend offered cigarette with warning** 1=More likely (5-7) (152), 0=More unlikely (1-4) (1418).		
	**N**	**AOR * (95% CI)**	**P-value**	**N**	**AOR * (95% CI)**	**P-value**	**N**	**AOR * (95% CI)**	**P-value**	**N**	**AOR * (95% CI)**	**P-value**	**N**	**AOR * (95% CI)**	**P-value**	**N**	**AOR * (95% CI)**	**P-value**
**Age**			**0.232**			**0.691**			0.199			0.984			0.972			0.798
18	195	ref		196	ref		228	ref		218	ref		267	ref		268	ref	
19	601	1.2(0.8,1.7)		631	1.1(0.8,1.6)		698	0.8(0.5,1.0)		700	1.0(0.7,1.4)		834	1.1(0.7,1.7)		830	0.9(0.5,1.5)	
At or above 20	382	0.9(0.6,1.4)		372	1.2(0.8,1.8)		405	0.7(0.5,1.1)		413	1.0(0.7,1.4)		473	1.0(0.6,1.7)		472	0.8(0.5,1.5)	
**Gender**			0.008			0.221			0.129			0.778			0.212			0.545
Female	684	ref		704	ref		808	ref		832	ref		945	ref		945	ref	
Male	494	1.5(1.1,1.9)		495	0.9(0.7,1.3)		523	1.2(1.0,1.5)		499	1.0(0.8,1.2)		629	1.2(0.9,1.7)		625	1.1(0.8,1.6)	
**Stream of Study**			0.856			0.111			0.042			0.322			0.396			0.116
Health science	430	ref		426	ref		472	ref		496	ref		563	ref		559	ref	
Others	352	1.0(0.7,1.4)		360	0.7(0.5,1.0)		404	0.7(0.5,0.9)		400	1.3(0.9,1.7)		492	1.0(0.7,1.5)		493	1.6(1.0,2.6)	
Technical	396	1.1(0.8,1.4)		413	1.0(0.7,1.3)		455	0.9(0.7,1.2)		435	1.0(0.8,1.4)		519	1.3(0.9,1.8)		518	1.2(0.7,1.9)	
**Religion**			0.037			0.511			0.362			0.558			0.023			0.111
Hindu	915	ref		929	ref		1044	ref		1034	ref		1235	ref		1232	ref	
Christian	159	1.3(0.9,1.9)		166	0.8(0.6,1.1)		174	1.3(0.9,1.9)		180	1.2(0.8,1.6)		212	0.6(0.4,0.9)		214	0.6(0.4,1.2)	
Muslim	67	0.5(0.2,0.9)		67	1.0(0.6,1.7)		73	1.0(0.6,1.6)		74	1.3(0.8,2.1)		85	0.8(0.4,1.4)		82	0.4(0.2,1.0)	
Others	37	1.2(0.6,2.5)		37	1.1(0.6,2.2)		40	0.8(0.4,1.6)		43	1.3(0.7,2.5)		42	1.8(0.9,3.7)		42	1.2(0.5,2.8)	
**Wealth quintiles**			0.309			0.407			0.037			0.259			0.002			0.122
Upper	206	ref		200	ref		208	ref		228	ref		251	ref		253	ref	

Upper Middle	181	0.9(0.6,1.4)		184	1.2(0.8,1.7)		201	1.1(0.8,1.7)		211	0.9(0.6,1.4)		233	1.0(0.6,1.5)		231	1.1(0.7,1.9)	
Middle	366	0.8(0.5,1.2)		374	1.2(0.8,1.7)		416	0.7(0.5,1.0)		394	1.1(0.8,1.6)		464	0.6(0.4,0.9)		462	0.6(0.4,1.0)	
Lower Middle	180	0.9(0.6,1.4)		190	1.0(0.6,1.5)		223	0.8(0.5,1.2)		217	1.3(0.9,2.0)		281	0.4(0.2,0.6)		281	0.6(0.3,1.1)	
Lower	245	0.6(0.4,1.0)		251	1.4(0.9,2.1)		283	0.7(0.4,1.0)		281	1.4(1.0,2.1)		345	0.6(0.3,0.9)		343	0.8(0.4,1.5)	
**Smoking status**			0.249			0.127			0.750			**0.005**			**<0.001**			**<0.001**
Ever	223	ref		217	ref		233	ref		242	ref		257	ref		259	ref	
Never	907	1.2(0.9,1.8)		934	1.4(0.9,2.1)		1047	1.1(0.8,1.4)		1045	1.6(1.2,2.2)		1266	0.2(0.2,0.3)		1261	0.3(0.2,0.4)	

* adjusted for all other variables in the model, AOR = adjusted odds ratio; ref = reference category; 95% CI, 95% confidence interval

© 2023 Mullapudi S. et al.
